# Pilot Preclinical and Clinical Evaluation of (4S)-4-(3-[18F]Fluoropropyl)-L-Glutamate (18F-FSPG) for PET/CT Imaging of Intracranial Malignancies

**DOI:** 10.1371/journal.pone.0148628

**Published:** 2016-02-18

**Authors:** Erik S. Mittra, Norman Koglin, Camila Mosci, Meena Kumar, Aileen Hoehne, Khun Visith Keu, Andrei H. Iagaru, Andre Mueller, Mathias Berndt, Santiago Bullich, Matthias Friebe, Heribert Schmitt-Willich, Volker Gekeler, Lüder M. Fels, Claudia Bacher-Stier, Dae Hyuk Moon, Frederick T. Chin, Andrew W. Stephens, Ludger M. Dinkelborg, Sanjiv S. Gambhir

**Affiliations:** 1 Molecular Imaging Program, Department of Radiology, Stanford Hospital and Clinics, Stanford, CA, United States of America; 2 Bayer Pharma AG, Berlin, Germany; 3 Piramal Imaging GmbH, Berlin, Germany; 4 Department of Nuclear Medicine, Asan Medical Center, University of Ulsan College of Medicine, Seoul, Republic of Korea; 5 Bio-X Program, Department of Bioengineering, Department of Materials Science & Engineering, Stanford University, Stanford, CA, United States of America; German Cancer Research Center (DKFZ), GERMANY

## Abstract

**Purpose:**

(S)-4-(3-[^18^F]Fluoropropyl)-*L*-glutamic acid (18F-FSPG) is a novel radiopharmaceutical for Positron Emission Tomography (PET) imaging. It is a glutamate analogue that can be used to measure x_C_^-^ transporter activity. This study was performed to assess the feasibility of 18F-FSPG for imaging orthotopic brain tumors in small animals and the translation of this approach in human subjects with intracranial malignancies.

**Experimental Design:**

For the small animal study, GS9L glioblastoma cells were implanted into brains of Fischer rats and studied with 18F-FSPG, the 18F-labeled glucose derivative 18F-FDG and with the 18F-labeled amino acid derivative 18F-FET. For the human study, five subjects with either primary or metastatic brain cancer were recruited (mean age 50.4 years). After injection of 300 MBq of 18F-FSPG, 3 whole-body PET/Computed Tomography (CT) scans were obtained and safety parameters were measured. The three subjects with brain metastases also had an 18F-FDG PET/CT scan. Quantitative and qualitative comparison of the scans was performed to assess kinetics, biodistribution, and relative efficacy of the tracers.

**Results:**

In the small animals, the orthotopic brain tumors were visualized well with 18F-FSPG. The high tumor uptake of 18F-FSPG in the GS9L model and the absence of background signal led to good tumor visualization with high contrast (tumor/brain ratio: 32.7). 18F-FDG and 18F-FET showed T/B ratios of 1.7 and 2.8, respectively. In the human pilot study, 18F-FSPG was well tolerated and there was similar distribution in all patients. All malignant lesions were positive with 18F-FSPG except for one low-grade primary brain tumor. In the 18F-FSPG-PET-positive tumors a similar T/B ratio was observed as in the animal model.

**Conclusions:**

18F-FSPG is a novel PET radiopharmaceutical that demonstrates good uptake in both small animal and human studies of intracranial malignancies. Future studies on larger numbers of subjects and a wider array of brain tumors are planned.

**Trial Registration:**

ClinicalTrials.gov NCT01186601

## Introduction

Primary and secondary brain tumors are of major medical importance as they almost always have very poor clinical outcomes. Primary brain tumors are relatively rare while secondary or metastatic brain tumors are more common and commonly arise from lung, breast, and skin (melanoma) cancers [1]. Therapeutic strategies include surgery, systemic chemotherapy, chemotherapy with impregnated biopolymers for local administration, whole-brain radiation, and radiosurgery [2–4].

The anatomic diagnostic modality of choice for the initial diagnosis and subsequent follow-up of brain tumors is magnetic resonance imaging (MRI) [5, 6]. However, MR has limitations, especially in the sensitivity of smaller lesions and in the specificity for the evaluation of the post-treatment brain [7]. Computed tomography (CT) has limited utility [8, 9]. Metabolic imaging with 18F-2-fluoro-2-deoxy-D-glucose (18F-FDG) positron emission tomography coupled with computed tomography (PET/CT), also has a limited role in the diagnosis of intracranial malignancy as the high physiologic 18F-FDG uptake in normal brain limits and reduces its sensitivity [10]. However, in the post-treatment setting, especially after radiation therapy, 18F-FDG PET can be used to distinguish between radiation necrosis and residual / recurrent tumor [10]. In an attempt to overcome some of these limitations with 18F-FDG, a variety of different amino acid-based radiopharmaceuticals have been investigated including methyl-11C-L-methionine (11C-MET), O-(2-18F-fluoroethyl)-L-tyrosine (18F-FET), 6-18F-fluoro-L-dopa (18F-FDOPA), and 18F-fluoro-L-thymidine (18F-FLT) [11], and most recently, 4-18F-(2S,4R)-fluoroglutamine (18F-FGln) [12].

Pre-clinical models and studies of intracranial malignancies are important to gain a better understanding on the underlying tumor biology and for the development and evaluation of novel diagnostic and therapeutic strategies [13]. For example, the interaction of migrating glioma cells with the preexisting vasculature outside the main tumor mass was examined in a murine glioma model recently [14] which may have clinical implications regarding blood flow in the tumor-associated brain and the ability to deliver therapeutics and diagnostics which usually not cross the BBB. Another important area of research is the metabolic adaptation of tumors on conditions of oxidative stress and the development of treatment resistance. Here the thiol-containing antioxidant glutathione, its precursors glutamate and cysteine as well as the transporter responsible for providing cystine as the sulfur component are crucial for tumors as described in more detail below.

The amino acid glutamate and its transporters play important roles in the brain [15]. Glutamate acts as neurotransmitter and maintaining low levels of extracellular glutamate is critically important for normal brain function. Hence, glutamate transporters are found on both neurons and glial cells to remove most of the extracellular glutamate to prevent aberrant glutamate signaling. Sodium-dependent excitatory amino acid transporters (EAAT) are the principal glutamate transporters responsible for extracellular glutamate homeostasis in the brain. In addition to EAAT glutamate transporters, a sodium-independent cystine-glutamate exchange transport system (system x_C_^-^) also influences extracellular glutamate levels. System x_C_^-^, a SLC7A11/SLC3A2 heterodimer, transports the amino acids *L*-cystine and *L*-glutamate. Intracellularly, *L*-cystine gets reduced to two molecules of *L-*cysteine. This represents an efficient way to get access to a rate-limiting precursor for glutathione biosynthesis and contributes to redox maintenance. The transporter acts by exchanging abundant intracellular glutamate for extracellular cystine or glutamate [16]. System x_C_^-^ is an emerging target in oncology, mediating oxidative stress and providing an additional growth advantage for a variety of tumors, including those in the brain. In the healthy brain system x_C_^-^ and EAAT complement each other by providing a glutamate gradient for system x_C_^-^ mediated cystine uptake and prevention of glutamate toxicity in the surrounding brain. Gliomas are mostly lacking EAAT transporters while showing increased glutaminolysis fueling the intracellular glutamate pool. The increased system x_C_^-^ activity for redox balancing and detoxification leads to accumulation of the released glutamate in the extracellular space causing a loss of glutamate homeostasis, tumor-associated seizures and neuronal death. This promotes enhanced survival, growth, migration and invasion of gliomas. Recently it was shown that expression levels of system x_C_^-^ are associated with seizures and can predict poor survival in patients with malignant glioma [17]. Also tumors from other origins exploit the system x_C_^-^ mechanism to provide cystine as precursor for the glutathione biosynthesis pathway from abundant intracellular glutamate and to mediate redox balance [16]. It is assumed that brain metastases derived from these tumors would still use this feature and would have a growth and survival advantage in the brain similar to gliomas as described before.

(4S)-4-(3-[^18^F]fluoropropyl)-*L*-glutamate (previously known as BAY 94–9392, and herein referred to as 18F-FSPG) is a novel 18F-labeled glutamate derivative for PET imaging. It is specifically transported via system x_C_^-^ as demonstrated recently in cell competition assays and SLC7A11 knock-down cells. Excellent tumor visualization was achieved in subcutaneous animal tumor models [18]. Biodistribution analysis in rodents showed a rapid blood clearance via the kidneys and low background activity (including in the brain) providing high contrast for tumor imaging [18]. First pilot clinical studies examining dosimetry and biodistribution in healthy volunteers [19] and tumor detection in non-small cell lung cancer, breast cancer, and hepatocellular cancer showed promising results [20, 21]. Radiosynthesis, preclinical data and tumor visualization using another radiolabeled system x_C_^-^ transporter substrate, ^18^F-5-fluoro-*L*-aminosuberic acid (18F-FASu), were described recently [22].

Given the importance of system x_C_^-^ in cancer biology and the promising preclinical and first clinical results of 18F-FSPG, particularly with low background uptake in the brain, the purpose of this study was to assess its level of tumor uptake in a preclinical orthotopic model of glioblastoma, as well as to perform the first-in-man studies of this radiotracer in patients with either primary or metastatic brain cancer.

## Methods

### Preclinical evaluation of 18F-FSPG

#### Radiochemistry

Cyclotron-produced 18F-fluoride was obtained in an aqueous solution after the 18O (p, n) 18F reaction. Radiolabeling of 18F-FSPG was accomplished by nucleophilic substitution of the nosylate precursor di-tert-butyl (*4S*)-N-(tert-butoxycarbonyl)-4-(3-{[(4-nitrophenyl) sulfonyl]oxy}-propyl)*-L*-glutamate using K^18^F kryptofix complex and subsequent acidic deprotection followed by cartridge purification. 18F-FDG for preclinical studies was obtained from Eckert & Ziegler Euro-PET GmbH (Berlin, Germany). 18F-FET was provided by IASON PET-Network (Graz, Austria).

#### In vivo animal studies

All animal experiments were performed in Berlin, Germany in compliance with the current local laws concerning animal protection and welfare and received local ethics committee approval (LaGeSo Berlin, permit number ANZ909). No animal studies were performed at Stanford University. All animals used in the studies were kept under normal laboratory conditions at a temperature of 22 ± 2°C and a dark/light rhythm of 12 hours. Food and water were provided *ad libitum*. Acclimation period was at least 7 days before the beginning of the studies. During this period, animals were examined to ascertain the absence of abnormal signs.

#### GS9L (rat glioblastoma) brain tumors in rats

To compare the imaging potential of 18F-FSPG, 18F-FDG and 18F-FET for brain tumor imaging, rat GS9L glioblastoma cells were orthotopically implanted into rat brains. For the evaluation of each tracer tumor cells were inoculated in 5 rats (Fisher F344, Charles River, male, 270–300g). Each PET tracer was evaluated sequentially in a different group of rats. Inoculation was performed under ketamine/xylazine anesthesia and all efforts were made to minimize suffering. A 2:1 mixture of ketamine (Ketavet®, 100 mg/ml, Medistar GmbH, Holzwickede, Germany) and xylazine (Rompun®, 20 mg/ml, Bayer Vital, Leverkusen, Germany) was administered i.p. at a dose of 1 ml/kg body weight. The head was placed and fixed according to standard procedures in a Kopf stereotaxic frame (Kopf Instruments). A Hamilton syringe was used to inject 5 μl of a GS9L cell suspension containing 1x10^6^ cells in PBS into the corpus callosum at the following coordinates: Bregma + 2.00 mm, lateral 2.0 mm and ventral 3.0 mm (the ventral coordinate refers to the surface of the brain). The presence of brain tumors was controlled after one week by MR measurements (data not shown).

#### Preclinical PET imaging

Each tracer was studied in a separate group due to the rapid tumor growth. All animals with confirmed presence of tumors received a tracer injection at day 9 after tumor implantation. The tracer uptake was quantitatively analyzed after sacrifizing the animals at study end (n = 3 animals for 18F-FDG; n = 4 animals for 18F-FET and n = 5 animals for 18F-FSPG). PET imaging was performed with 3 animals for each tracer using the Inveon small animal multimodality PET/CT scanner (Siemens, Knoxville, TN, USA). 100–500 μL tracer solution containing an activity of 15 MBq was injected via tail-vein into isoflurane (Abbot) anesthesized rats. PET data obtained in this study were acquired in a dynamic mode and were analyzed time-resolved afterwards. The dynamic PET measurement was started for 30 minutes directly after tracer injection. After finishing the PET study, the animals were sacrificed approximately 45 min post injection while still anesthetized, and the tumor and other organs of interest were removed, weighed and radioactivity determined using a gamma-counter. Tracer uptake was calculated as the percentage of injected dose per gram of tissue (% ID/g). The mean % ID/g value and the standard deviation were calculated for each time point. PET data were reconstructed using the OSEM-2D algorithm. Images were qualitatively and quantitatively analysed with the IRW software provided by the manufacturer. The area of tumor cell inoculation was visually assessed for tracer uptake. A 3D volume-of-interest (VOI) was manually drawn to cover the tumor lesion for quantification of uptake and kinetic analyses. An equally sized VOI in the healthy brain tissue was used to normalize the data by dividing the tumor uptake by the normal brain uptake to create a tumor-to-normal brain ratio. This normalization step was performed for each rat and the resulting averaged time-activity curves were plotted for comparing each tracer.

### Clinical evaluation of 18F-FSPG

#### Radiochemistry

Clinical grade 18F-FSPG was prepared via nearly the same synthetic procedure as preclinical grade 18F-FSPG. The only exception is the use of tetrabutylammonium hydrogen carbonate (TBAHCO3) instead of K2CO3/kryptofix. The fully automated synthesis was performed in a Modular-Lab radiosynthesizer from Eckert & Ziegler Euro-PET GmbH (Berlin, Germany) and took 75 min from end of bombardment to achieve final product. Clinical grade 18F-FDG was supplied by the Radiochemistry Facility of the Molecular Imaging Program at Stanford University.

#### Human imaging studies

**Fig 1:**
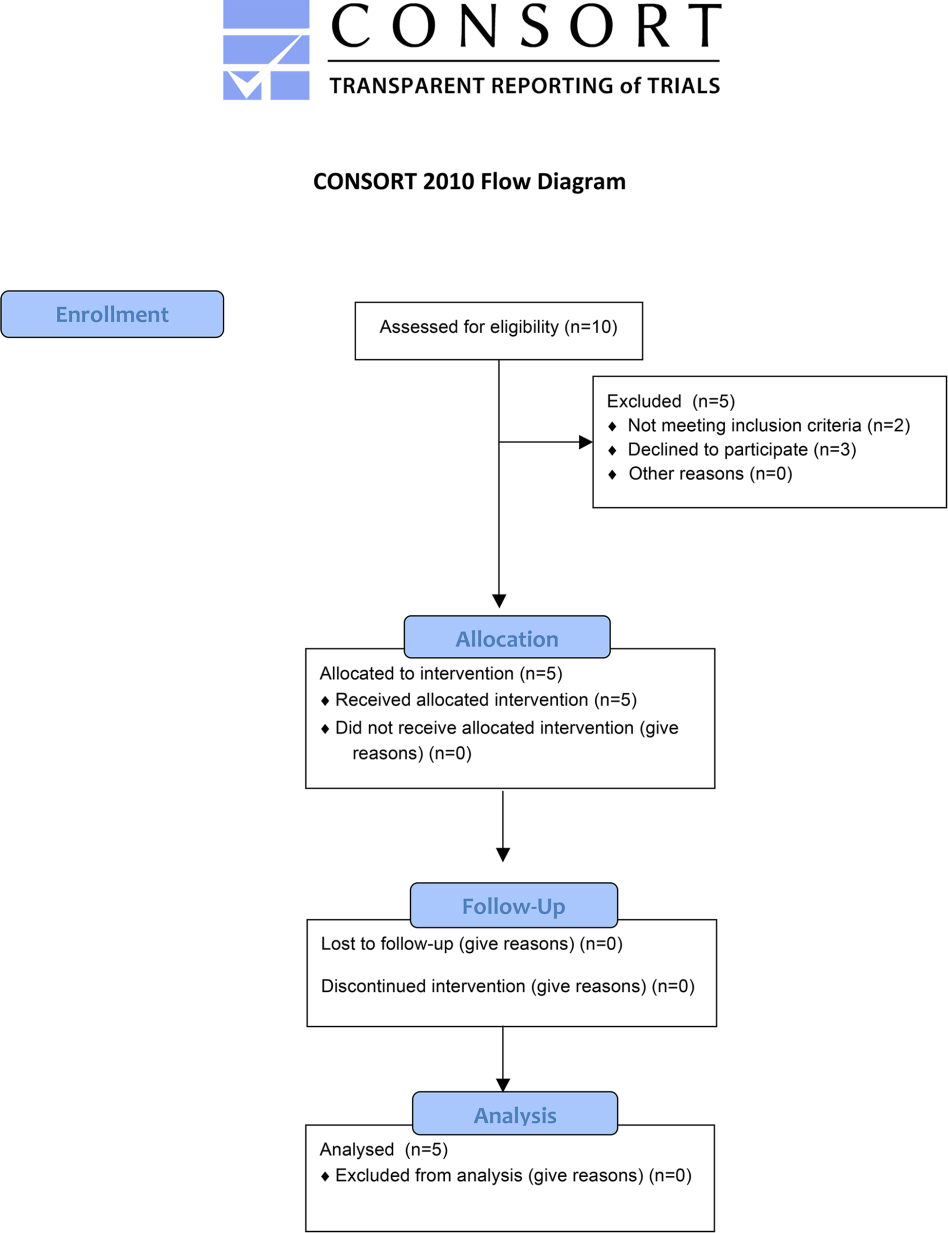
CONSORT Participant Flow Diagram.

The Institutional Review Board (IRB) at Stanford University, the Scientific Review Committee (SRC) at the Stanford Cancer Institute, and the US Food and Drug Administration (eIND 108509) approved the clinical study protocol (see S2 Table for inclusion/exclusion criteria). This study is part of a larger multinational umbrella exploratory program evaluating the safety, tolerability, dosimetry and potential ability to image a variety of tumors. Safety data from all centers will be compiled and reported at a later date. From April—October 2011, five patients with primary or secondary brain tumors were recruited for an 18F-FSPG PET/CT (Fig 1).

Before the PET/CT scan was performed, a physical exam, a 12-lead electrocardiogram (ECG), vital signs (blood pressure, heart rate, body temperature), blood sampling (for complete blood chemistries, comprehensive metabolic panel, international normalized ratio, and activated partial thromboplastin time) and urine (for urine analysis and pregnancy test, if needed) were also collected. Routine medications were also recorded. A dose of 18F-FSPG with a total mass dose of ≤ 100 μg was administered as a slow intravenous bolus injection over 60 seconds. The mean activity was 298.29 MBq, range 244.20–327.08 MBq. After the tracer injection, the cannula and injection system was flushed with 10 mL normal saline (0.9% NaCl).

Three whole-body PET/CT scans were then acquired sequentially to capture different time points after tracer injection. The images were obtained using a GE Discovery 600 or 690 PET/CT scanner (GE Healthcare, WI, USA). The first scan, with a total duration of 45 minutes, was done immediately after the injection of tracer. It was performed as five sequential whole-body (vertex to mid-thigh) PET acquisitions after obtaining one CT (140 kV, range 10–85 mA) for attenuation correction and anatomic localization. Each of these 5 PET acquisitions increased the number of minutes per bed position as follows: 30s/bed, 30s/bed, 1min/bed, 2min/bed, and 2min/bed. The second and third whole-body PET/CT scans, each with a duration of approximately 30 minutes (3 min/bed position), were started 60 and 105 minutes post injection, respectively. The patients were also asked to void between each scan in order to reduce the total radiation exposure.

After the scans were complete, another set of ECG, vital signs, and blood and urine samples were collected, as well as recording any adverse events either noted by the members of the research team or the participants. Within 24 hours of the scan, the participants returned to the clinic and had repeat physical examination, ECG, vital signs, and blood and urine sampling done. Seven days later, the patients were contacted by phone to know about any interim adverse events or medication changes.

The three patients with metastatic lung cancer also had standard-of-care 18F-FDG whole-body PET/CT scans done. These were performed on the same scanners indicated above, an average of 11 days before the 18F-FSPG PET/CT scan. The technical details of these routine scans have been described elsewhere [23, 24].

#### Analysis of the clinical 18F-FSPG images

Both the 18F-FSPG and 18F-FDG PET/CT scans were evaluated by three nuclear medicine physicians (ESM, MK, CM) using a GE Advanced Windows (AW) workstation. These physicians were not blind to the subject’s clinical data and correlative imaging findings (if any). Standardized uptake values (SUV) were rigorously measured on all scans (i.e., at all time points to track the time-activity relationship of the tracer) using the GE AW workstation. Different types of 2D regions of interest (ROI) or 3D volumes of interest (VOI) were used depending on the organ or region of interest, but applied consistently across all scans as follows. VOIs with a volume of 19.08 cm^3^, were drawn on the transaxial PET scan over the brain, left ventricle of the heart, right lung parenchyma, right hepatic lobe, left gluteal musculature, the kidney cortex and both primary and metastatic lesions. Based on visual estimation, the VOI size was adjusted for the actual lesions so that only the lesion, and no surrounding structures, was captured. ROIs, with an area of 387.1 mm^2^, were drawn over the aortic arch. An irregular VOI using the region growth algorithm, visually adjusting the boundary threshold as needed to include as much of the organ of interest as possible without including adjacent structures, was utilized for the whole kidney and pancreas.

Both the SUV maximum and the SUV mean were measured for each structure as well as the bidimensional size of each lesion from CT. SUV was calculated using the formula SUV = decay-corrected radioactivity in a region of interest/(injected dose/patient’s body weight).

### Statistical analysis

The three radioligands used in the preclinical study were compared to each other using the Kruskal-Wallis test in terms of their absolute blood, brain, and tumor uptake as well as their tumor-to-brain and tumor-blood ratios. Additional pairwise multiple comparison testing was performed using the Nemeny post-hoc test with the PMCMR package. P-values <0.05 were considered as statistically significant.

To assess changes in a given lab value and vital sign from baseline, 3 hours post-tracer injection and at day 1 post-tracer injection, statistical analysis was performed using a repeated measures ANOVA. The ANOVA tests were conducted using R, version 3.0.1 (www.r-project.org).

## Results

### Pre-clinical studies

#### Comparative PET imaging of GS9L Glioblastoma bearing rats with 18F-FSPG, 18F-FDG and 18F-FET

**Fig 2:**
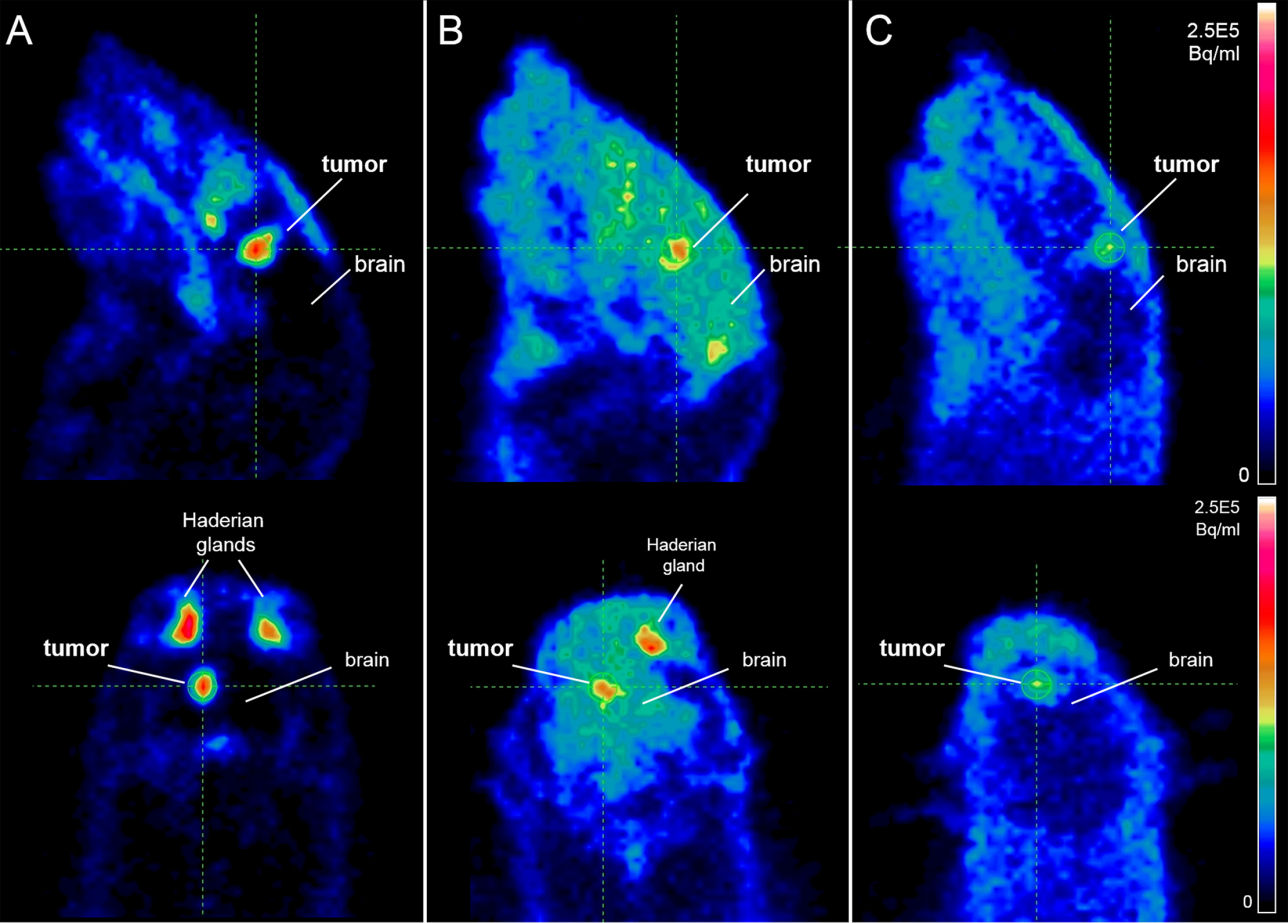
**PET imaging of rat GS9L brain tumors with (A) 18F-FSPG (B) 18F-FDG and (C) 18F-FET.** Selected sagittal (upper images) and coronal (lower images) slices are shown (dose: 15 MBq each, imaging time: 0–30 min p.i.).

**Fig 3:**
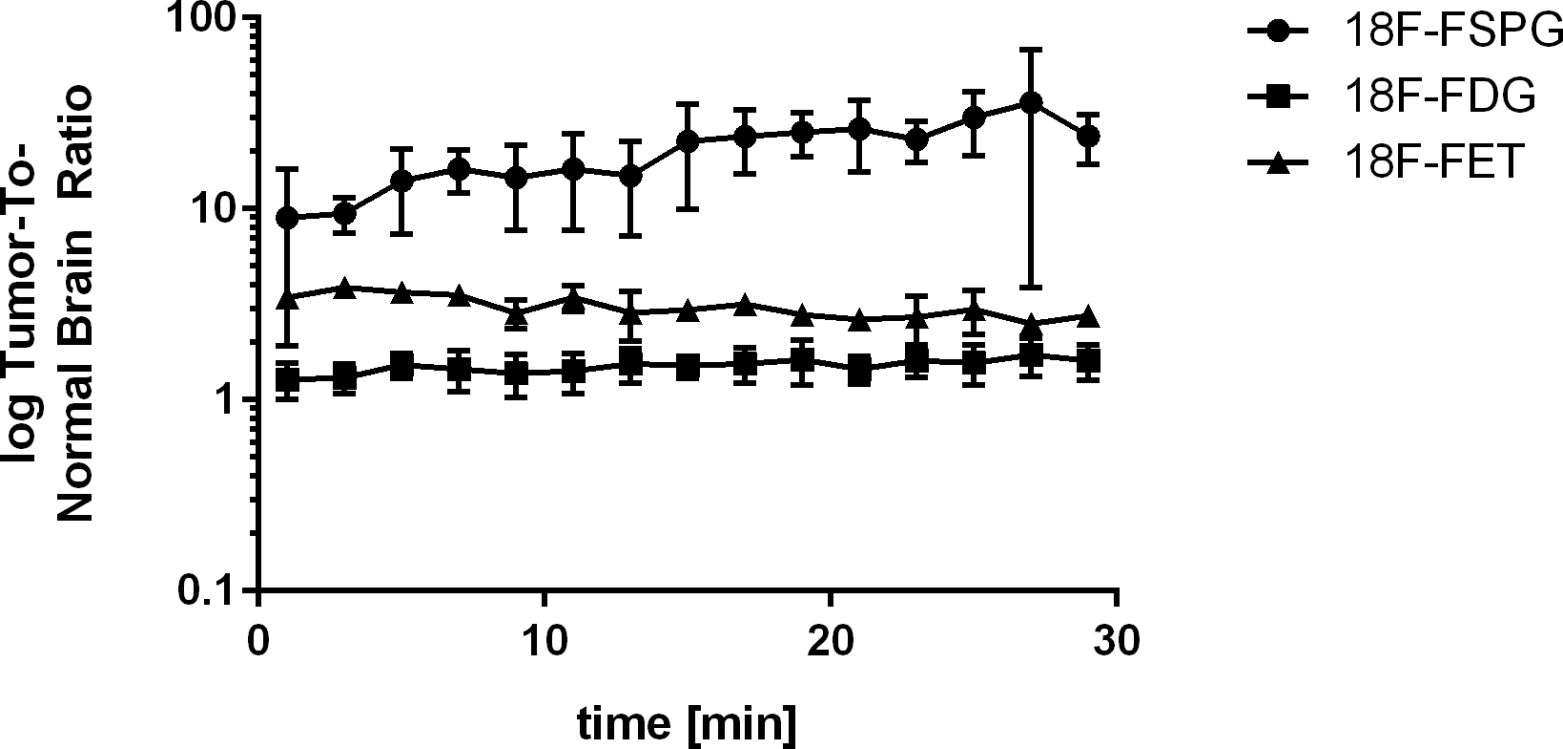
Time-activity curves of 18F-FSPG, 18F-FDG and 18F-FET tumor-uptake ratio in orthotopic GS9L rat brain tumor lesions. A volume of interest (VOI) analysis over time was performed from the tumor lesions and the ratio calculated using the VOI from the healthy brain for each tracer. 18F-FSPG showed increasing VOI ratio activity over time. In contrast, 18F-FDG and 18F-FET VOI ratios remained rather constant and at a lower level.

Orthotopic brain tumor visualization was possible with 18F-FSPG, 18F-FDG, and 18F-FET in a rodent model (Fig 2). The high tumor uptake of 18F-FSPG and the absence of background signal in the healthy brain led to good tumor visualization with high contrast. Conversely, tumor visualization with 18F-FDG is compromised due to the high uptake of the tracer in the surrounding healthy brain. 18F-FET can be used to visualize the GS9L tumors, but it showed a lower overall tumor uptake compared to the other tracers investigated in this model. Strong tracer uptake in the Harderian glands, a rodent-specific gland, was observed for 18F-FSPG and 18F-FDG but not for 18F-FET. These glands are located behind the eyes and play a role in porphyrine biosynthesis in rodents and cats but not in humans. Time-activity curves of the tumor regions were generated and normalized to a region of normal brain. The tumor-to-normal brain ratios for all three tracers during the first 30 min post injection are shown in Fig 3. There was a higher tumor-to-normal brain ratio for 18F-FSPG over both 18F-FET and 18F-FDG.

Table 1 shows quantitative ex vivo data from all tumor-bearing animals investigated in this study. A tumor-to-normal brain ratio of 32.7 was obtained with 18F-FSPG. The absolute tumor uptake of 18F-FSPG was comparable to 18F-FDG, but the higher normal brain signal of 18F-FDG led to a tumor-to-normal brain ratio for 18F-FDG of only 1.7. 18F-FET showed a lower absolute tumor uptake with a moderate degree of uptake in the normal brain as reflected in a moderate tumor-to-normal brain ratio of approximately 2.8. Tumor to normal brain ratios derived from the PET study (at 30 min post injection) and ex vivo measurement at approximately 1 hr post injection are in good agreement. Significant differences between the three radiotracers were observed by using the Kruskal-Wallis test and are shown in S1 Table.

**Table 1:** Comparison of 18F-FSPG, 18F-FET and 18F-FDG uptake in rat GS9L brain tumors, healthy brain and blood at approximately 1 hr post injection. The tracer uptake was measured ex vivo and is shown as % ID/g values with standard deviation and ranges. Tumor-to-normal brain (T/brain) and tumor-to-blood ratios (T/blood) were derived from the respective mean values.

Organ	18F-FSPG	18F-FDG	18F-FET
	[mean (SD)], range	[mean (SD)], range	[mean (SD)], range
**Tumor** (% ID/g)	1.64 (0.61), 0.97–2.29	1.55 (0.35), 1.32–1.95	0.65 (0.20), 0.48–0.90
**Brain** (% ID/g)	0.05 (0.03), 0.02–0.09	0.92 (0.23), 0.72–1.18	0.23 (0.04), 0.20–0.28
**Blood** (% ID/g)	0.15 (0.06), 0.10–0.25	0.29 (0.01), 0.28–0.30	0.49 (0.02), 0.46–0.50
**T/brain ratio**	32.7	1.7	2.8
**T/blood ratio**	10.8	5.3	1.3

### Clinical studies

#### Evaluation of 18F-FSPG PET/CT in patients with primary and secondary brain tumors

Two men and 3 women were recruited with the full demographics shown in Table 2. None of the five subjects had any adverse events throughout the duration of their participation in the clinical trial, either in terms of self-described symptoms, or vital signs or laboratory values. As noted in S3 Table, from the measured safety and lab values only the systolic blood pressure showed a significant difference (p-value = 0.03) between the three time points.

**Table 2:** Demographic information for the 5 human subjects imaged with 18F-FSPG.

Subject	Sex	Age	Pathology	Prior treatment (months before)	18F-FSPG Dose (MBq)
1	F	29	Grade 2 oligodendroglioma	Subtotal resection (11)	327
2	F	45	Grade 4 glioblastoma	Gamma knife (4)	304
3	M	64	Metastatic NSCLC	Cyberknife (7)	244
4	F	49	Metastatic NSCLC	Chemotherapy (1)	312
5	M	65	Metastatic NSCLC	-	303

**Fig 4:**
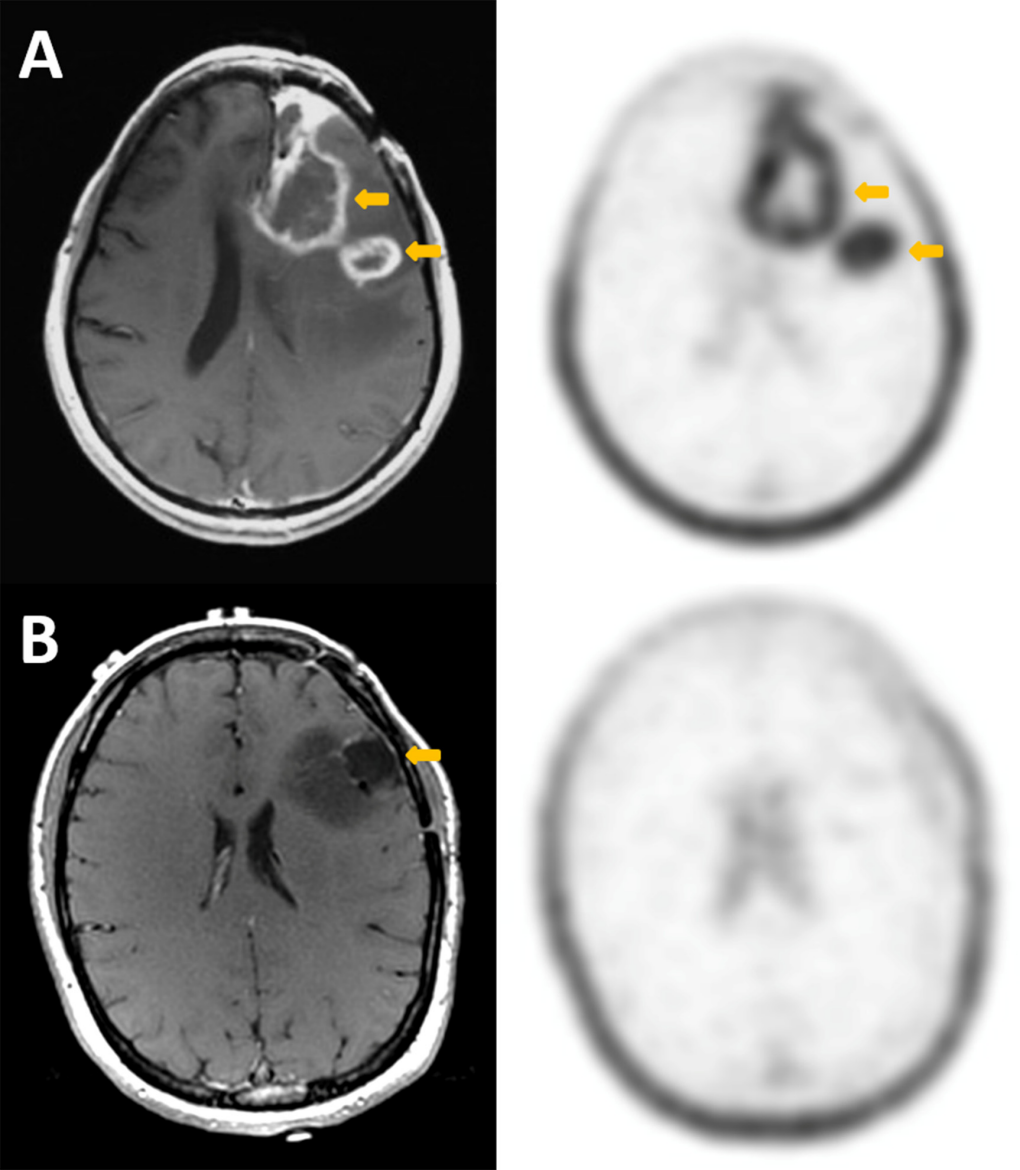
Comparison of axial post-contrast T1 MRI (left column) and 18F-FSPG PET (right column) in the two subjects with primary brain tumor. Subject A has recurrent high-grade glioblastoma, while subject B has an enlarging, partially resected low-grade oligodendroglioma. In each case, the arrows point to the primary lesions. For subject A, there is intense accumulation of 18F-FSPG in the enhancing lesion (SUV 5.2), while in subject B, there is no accumulation of the radiotracer (SUV 0.7) in the non-enhancing lesion. Comparatively, the background brain SUV is 0.1 for both subjects. There is incidental note of prominent physiologic uptake of 18F-FSPG in the scalp.

All subjects had very low uptake in the background normal brain parenchyma (SUVmax 0.1–0.2). The two subjects with primary brain tumor imaged for this study had different pathologies (Fig 4). The first had a high-grade glioblastoma multiforme (GBM) with two lesions in the left frontal lobe, which had previously been treated with gamma knife radiosurgery several months before. An MRI performed 11 days prior to the 18F-FSPG scan showed enhancement in the areas of prior disease, which was equivocal for residual/recurrent malignancy versus post-treatment inflammation. Clinically, the patient was having increased seizure activity. The 18F-FSPG scan in this patient shows intense uptake in both lesions (average SUVmax 4.6, range 3.9–5.2). No rebiopsy of the lesions were done to confirm the findings, but the patient was restarted on prednisone and scheduled for additional radiosurgery based on clinically presumed recurrence.

The second subject imaged with primary brain tumor had low-grade oligodendroglioma also in the left frontal lobe. She had a subtotal resection 11 months prior to the 18F-FSPG PET/CT. Serial MRI scans in the interval suggested disease progression, primarily based on increasing size of the tumor, but did not show any significant enhancement. In this case, the 18F-FSPG shows no uptake in the tumor. The next day, this patient had a craniotomy and additional resection of the tumor. Presence of oligodendroglioma was confirmed histologically.

**Fig 5:**
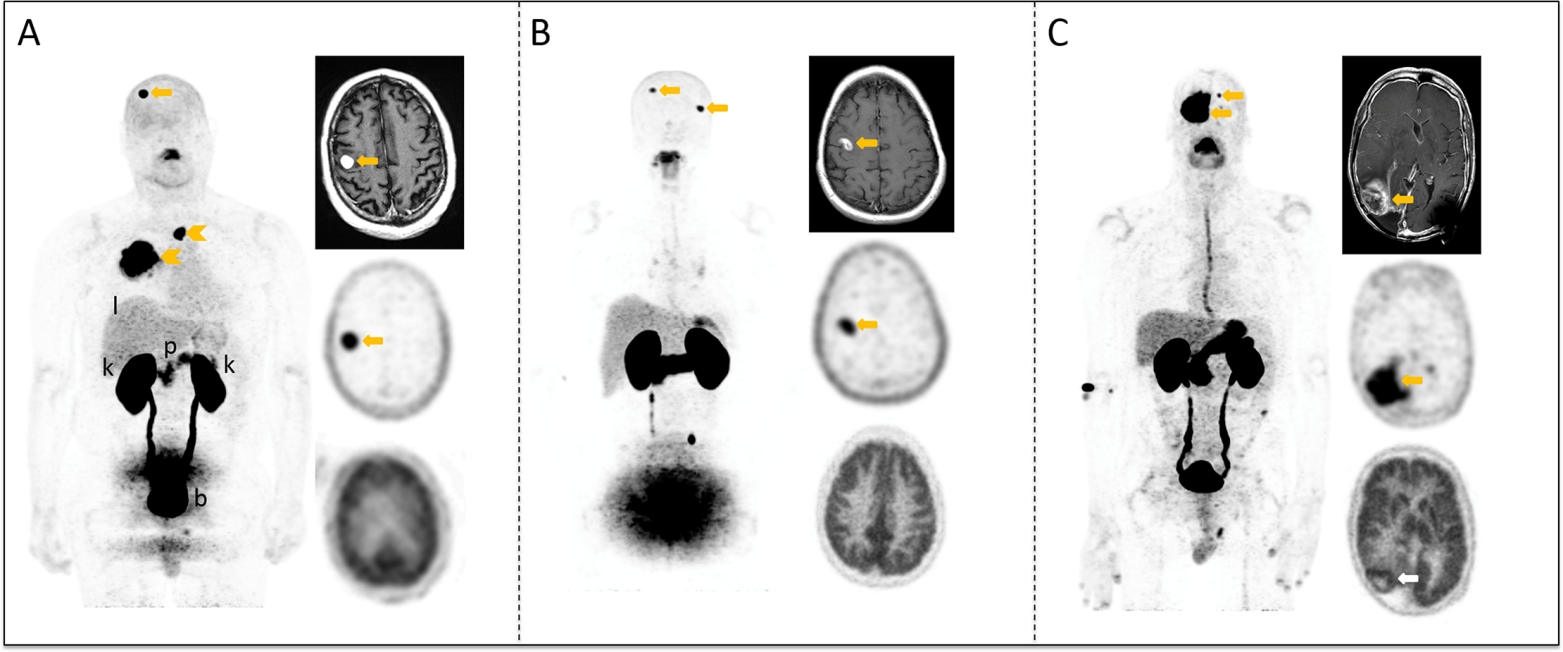
18F-FSPG PET images, in comparison with MRI and 18F-FDG PET images of the three patients with lung cancer metastases to the brain. For each subject, the whole-body Maximum Intensity Projection (MIP) image of the 18F-FSPG PET scan is shown on the left. In the right column, the axial images through the level of the brain metastasis include the post-contrast T1 MRI (top), 18F-FSPG PET (middle), and 18F-FDG PET (bottom). Physiologic distribution to normal organs are highlighted on the MIP image for subject A, including the liver (l), pancreas (p), kidneys (k), and bladder (b). For the first two subjects (A, B), the small lesions (below 1.5 cm) are clearly visible on MRI and with 18F-FSPG (SUV-A: 11.0, SUV-B: 4.7). With 18F-FDG, however, there is no discernible activity in these lesions. The larger lesion for subject C, who had previously been treated in this region, is again clearly discernable with MRI although the etiology of the enhancement was unclear whether representing residual/recurrent disease versus post-therapy changes. Both the 18F-FDG (SUV 10.1) and 18F-FSPG PET (SUV 21.8) are positive for this subject, but the accumulation of the latter is stronger, and further enhanced by the lack of uptake in the surrounding normal brain tissue (FDG SUV 4.8, FSPG SUV 0.1).

The three subjects with metastatic non-small cell lung cancer (NSCLC) to the brain all showed high 18F-FSPG uptake (average SUVmax 11.1 ± 7.6, range 4.7–21.8) in the brain metastases despite relatively small lesions in two of the three cases (Fig 5). The average lesion size for each of these patients is 1.3 cm, 1.7 cm, and 3.4 cm, respectively. On the comparative 18F-FDG scan, the first two subjects showed no uptake above background, while the third subject showed mild uptake (SUVmax 10.0) above background grey matter (SUVmax 9.0).

S1 and S2 Figs show the time-activity curves for 18F-FSPG across various organs and in the brain lesions for all 5 subjects. The 18F-FSPG signal in the healthy organs decreases over time with the exceptionof the kidneys and pancreas. The activity in the kidneys increases over the first 15 minutes then decreases, as the tracer is cleared through the kidneys. The signal from the pancreas slowly increases over the 105 minutes of imaging. The background brain uptake is very low, essentially zero, throughout the timecourse of imaging. S2 Fig shows the steady increase in activity within the brain lesion until 30 minutes, then essentially stabilizing through 105 minutes. Table 3 shows the comparison of the 18F-FSPG and 18F-FDG SUV values, including tumor-to-background ratios, at 60 minutes post-injection.

**Table 3:** Comparison of the SUV mean values at 60 minutes post-injection for 18F-FSPG and 18F-FDG across all patients (N = 5).

SUV 60 min p.i. (g/ml)	18F-FSPG [mean (SD)]	18F-FDG [mean (SD)]
**Primary brain tumors**	2.4*	NA
**Brain metastases**	5.0 (1.8)	7.2 (2.8)
**Aorta**	0.8 (0.2)	-
**Pancreas**	5.9 (2.1)	-
**Heart**	0.7 (0.1)	-
**Kidney whole**	10.4 (4.1)	-
**Kidney cortex**	9.5 (3.2)	-
**Brain**	0.1 (0.0)	6.9
**Muscle**	0.2 (0.1)	-
**Lung**	0.2 (0.1)	-
**Liver**	1.4 (0.3)	-
**Brain tumor:normal brain ratio**	24.0	-
**Brain mets:normal brain ratio**	50	1.0

*Average of the two lesions from the high-grade glioma subject.

S3 Fig shows the individual time activity curves for the extra-cranial lesions encountered for the two subjects with NSCLC that had them. Subject 3 had active malignancy in his primary lung cancer on the right, as well as thoracic vertebral body metastases, both of which show increasing 18F-FSPG activity throughout the time-course of imaging. Conversely, Subject 4 had an inactive/treated primary lung lesion in the left upper lobe, and reactive lymphadenopathy in the bilateral mediastinal and hilar regions, which show decreasing activity throughout the time-course of imaging.

## Discussion

The purpose of this study was to assess the ability of 18F-FSPG for brain tumor detection in a preclinical model of glioblastoma, as well as in humans with either primary or secondary (metastatic) brain tumors. Notably different to other amino acid based PET probes currently investigated in this setting that are taken up by ASCT2 or L-type amino acid transporter (LAT) systems, 18F-FSPG is taken up by system x_C_^-^ which defines a different metabolic phenotype. Visualization of PET tracer uptake via ASCT2 or LAT reflects nutrient uptake to cope with increased biomass and energy demands during proliferation. In contrast, 18F-FSPG uptake via system x_C_^-^ is associated with cellular responses to oxidative stress and detoxification processes which are important for tumor progression and resistance to treatment (Table 4).

**Table 4:** Overview of amino acid transporters being studied clinically using various PET tracers.

Amino Acid Transporter Type	SLC nomenclature of transporter subunit conferring substrate specificity	Tracer(s) currently investigated clinically	Main transporter function
ASCT2	SLC1A5	18F-Gln	Nutrient supply
LAT1 and/or LAT2	SLC7A5 / SLC7A8	11C-Met, 18F-FDOPA, 18F-FET	Nutrient supply
System x_C_^-^	SLC7A11	18F-FSPG	Redox balance, detoxification

The rat GS9L orthotopic brain tumor model evaluated in this study shows the potential utility of 18F-FSPG compared to 18F-FDG and 18F-FET under the conditions used. While both 18F-FSPG and 18F-FDG show good uptake in the tumor itself, the low background uptake in the surrounding normal brain for 18F-FSPG versus the high background for 18F-FDG, results in very favorable tumor-to-background (T/B) ratios for 18F-FSPG compared to 18F-FDG. 18F-FET PET showed more modest uptake in the primary tumor. Despite the small sample size, significant differences were observed between 18F-FSPG & 18F-FDG and 18F-FSPG & 18F-FET but not between 18F-FDG & 18F-FET. Of note, no restriction to food was applied for the animals prior to PET imaging as also described previously for 18F-FSPG [18] and 18F-FET [25, 26].

The promising results seen in the preclinical setting were also observed in the human subjects with primary and metastatic brain cancer. No significant differences in safety and lab parameters were observed after tracer injection except a slight increase in the systolic blood pressure. The low absolute rise in this value is unlikely to be of clinical significance and likely attributed to the activities associated with the injection and scans. The first patient with a primary brain tumor (recurrent high-grade GBM after chemo and radiation therapy), showed high 18F-FSPG uptake in both her lesions. This patient’s MRI findings were equivocal in the post-therapy setting, but the 18F-FSPG findings were consistent with the patient’s worsening clinical features. The second patient with primary brain tumor, a low-grade oligodendroglioma after partial resection 11 months prior, showed essentially no uptake of 18F-FSPG. Although the patient’s MRI showed no contrast enhancement, FLAIR abnormalities and increase in size were suggestive of residual disease. The patient had craniotomy with additional surgical resection the day after her 18F-FSPG scan which confirmed, residual malignancy surrounding the resection cavity. These two subjects with primary brain tumors show, albeit in a limited manner, the variability of uptake when imaging system x_C_^-^ activity with 18F-FSPG. In the face of the equivocal MRI scans for the first subject, the intense 18F-FSPG uptake suggests that this radiotracer can differentiate residual/recurrent high-grade malignancy from evolving post-therapy changes. This is a potentially valuable application for this novel radiotracer. The second subject potentially shows the ability of 18F-FSPG to discriminate between low-grade and high-grade tumors. Additionally, this may suggest that the accumulation of 18F-FSPG in the brain is specific to tumor cells with increased system x_C_^-^ activity which is also supported by the literature [27]. Recently, a novel metabolic pathway was discovered of cysteine catabolism contributing to glioblastoma growth [28]. Here, the metabolite cysteine sulfinic acid (CSA) was identified as one of the top metabolites differentiating glioblastoma from low-grade glioma which further supports the hypothesis that upstream system x_C_^-^ activity is associated with high-grade tumors.

All three subjects with metastatic NSCLC to the brain, showed high uptake of 18F-FSPG in their brain metastases. These findings are promising for two reasons. First, the lesions for two of these patients were relatively small (less than 1.5 cm), yet had intense uptake of 18F-FSPG. Secondly, each of these patients had a comparative 18F-FDG scan that showed either no or minimal activity above background in the same lesions. The latter is not surprising in itself as 18F-FDG PET is not known to be of particular utility in the evaluation of metastatic brain tumors due to high background uptake of 18F-FDG in normal brain tissue [10]. However, that these same lesions were so clearly evident on the 18F-FSPG scan supports its utility over 18F-FDG. An additional interesting finding in these subjects was that 18F-FSPG was able to clearly differentiate malignant from non-malignant extra-cranial lesions. The subject (3) who had an active lung malignancy and bone metastases showed increasing time-activity-curves while another subject (4) who had a treated primary lung lesion and reactive mediastinal lymphadenopathy showed decreasing time-activity-curves.

18F-FSPG uptake is mediated by the x_C_^-^ transporter. High transporter mRNA levels have been described in most brain regions [29, 30]. It is also known that malignant glioma cells release glutamate through the x_C_^-^ transporter, both to kill surrounding cells and to promote malignant cell invasion [15, 31]. Conversely, the x_C_^-^ transporter has not been described in an intact BBB. This likely explains the low uptake of 18F-FSPG in normal brain parenchyma.

The results of this pilot clinical study are promising, but should be taken with caution given the small sample size. However, the results suggest that additional work is warranted with a larger number of subjects and in a variety of intracranial malignancies. Other tracers beyond 18F-FDG currently used for brain tumor imaging such as the amino acid derivatives 11C-MET, 18F-FET, 18F-FDOPA and 18F-FGln are able to cross the BBB. The implication for 18F-FSPG for relying on a disrupted BBB for lesion imaging needs to be further elaborated while it ensures very low background signal from normal brain parenchyma. A background SUVmean of 0.1 was observed for 18F-FSPG in this study (Table 3). Recently, a study was published where 18F-FET and 18F-FDOPA were studied in the same patients and a background SUVmean of 1.0 was observed for 18F-FET and 1.5 for 18F-FDOPA. [32]. Of note, high lesion uptake has been observed for 18F-FSPG in this study with SUVmax values of up to 20 and SUVmean T/B ratios of >30. Other amino acid based PET tracers show generally lower lesion uptake and higher normal brain uptake resulting in T/B ratios of 4–5 (18F-Gln) [12], 3.4–4.3 (18F-FET) and 3.3–3.6 (18F-FDOPA) [32].

Potential future applications for 18F-FSPG PET may include as a diagnostic aid to MRI for diagnosis and treatment planning, evaluation of recsidual / recurrent malignancy, and for surveillance. Given the high uptake of 18F-FSPG in even small brain metastases and its favorable whole-body biodistribution pattern and uptake in tumors from various primary malignancies, the traditional whole-body staging PET scan could be adjusted with this tracer to include the brain and thereby identify many, if not all, of these lesions. This could have a potential impact on the imaging algorithm for staging of various malignancies. In the post-therapy state, MRI is often unable to differentiate between residual/recurrent malignancy and post-therapy related changes/inflammation. 18F-FDG PET has been used in this arena, but usually shows only very slightly increased uptake over the normal brain background, making the scans hard to evaluate and thereby reducing the sensitivity. 18F-FSPG, with its high T/B ratio could potentially be used at even earlier timepoints after therapy thereby affecting patient management and outcomes sooner.

Another area with potential impact as now both the metabolic and anatomic information can be used together, as is done for other tumors, is to help delineate the target volume for radiation treatment planning. Similarly, the combined information can be used for biopsy planning as well. Additional information on the underlying metabolic phenotype and adaptations against oxidative stress may provide a better understanding of the underlying tumor biology and chemoresistance mechanisms and would be useful for therapy selection and monitoring.

## Conclusions

18F-FSPG is a novel PET radiopharmaceutical that is specifically taken up by system x_C_^-^ as demonstrated by various preclinical tumor models and in orthotopically implanted GS9L brain tumors in rats, with low-background in healthy tissues. These findings were then translated to the clinic, where five patients with primary brain tumors or metastatic brain cancers were imaged. All the metastatic lesions to the brain were clearly identified, as well as a recurrent high-grade primary glioblastoma. There was no uptake in one subject with low-grade oligodendroglioma. Future studies based on larger numbers of subjects and those with a wider array or primary and secondary brain tumors are planned to further evaluate the utility of this promising new tracer.

## Supporting Information

S1 Checklist TREND Checklist. (PDF)

S1 Fig Time-activity curves of 18F-FSPG (SUV mean in g/ml) for various normal organs for all five subjects. Each data point represents the mean value with standard deviations across all subjects. There is a gradual decrease in activity in all normal organs except for the pancreas. The values are decay-corrected.
(TIFF)

S2 Fig Time-activity curves of 18F-FSPG (SUV mean in g/ml) for primary and metastatic brain tumor lesions from all five patients. The brain lesions show a gradual increase in activity until 30 minutes then decreasing minimally. The only exception is Subject 2 with the low-grade oligodendroglioma who had no uptake.
(TIFF)

S3 Fig Time-activity curves of 18F-FSPG (SUV mean in g/ml) for various non-brain primary and metastatic lesions for two patients who had them. The active primary lung tumor and bone metastases for Subject 3 showed continually increasing activity over the time-course of imaging. The treated primary lung tumor and inflammatory lymph nodes for Subject 4 showed decreasing activity throughout the time-course of imaging.
(TIFF)

S1 Protocol Study Protocol. (PDF)

S1 Table The comparison of the 18F-FSPG, 18F-FET and 18F-FDG uptake in blood, normal brain and brain tumor as well as analyses of tumor-to normal brain and tumor-to-blood ratios. Significant differences are marked in bold.
(DOCX)

S2 Table Specific inclusion and exclusion criteria for enrollment in the human 18F-FSPG PET/CT imaging trial. (DOCX)

S3 Table Average and standard deviation (S.D.) values for subjects’ vital signs, blood hematologies, and blood chemistries before injection of 18F-FSPG, after completion of imaging (approximately 3 hours post-injection), and the next day (approximately 24 hours post-injection). (DOCX)
