# Supplementary figures and images for: Pilot Preclinical and Clinical Evaluation of (4S)-4-(3-[18F]Fluoropropyl)-L-Glutamate (18F-FSPG) for PET/CT Imaging of Intracranial Malignancies
Source: PLoS One. 2016 Feb 18;11(2):e0148628. doi: 10.1371/journal.pone.0148628 (PMC4758607; doi:10.1371/journal.pone.0148628)

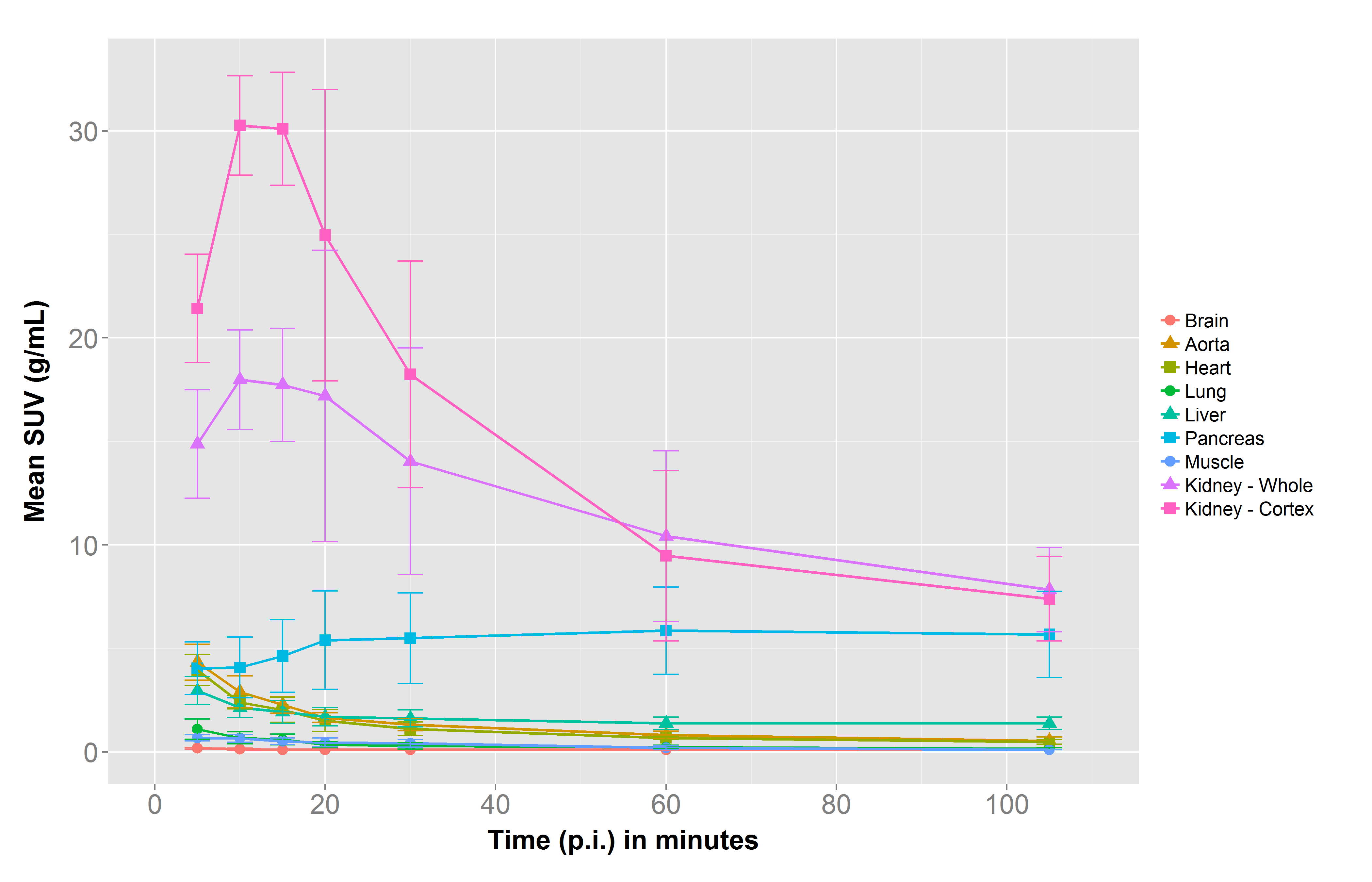

Supplement: S1 Fig — Each data point represents the mean value with standard deviations across all subjects. There is a gradual decrease in activity in all normal organs except for the pancreas. The values are decay-corrected. (TIFF) [file pone.0148628.s002.tiff]

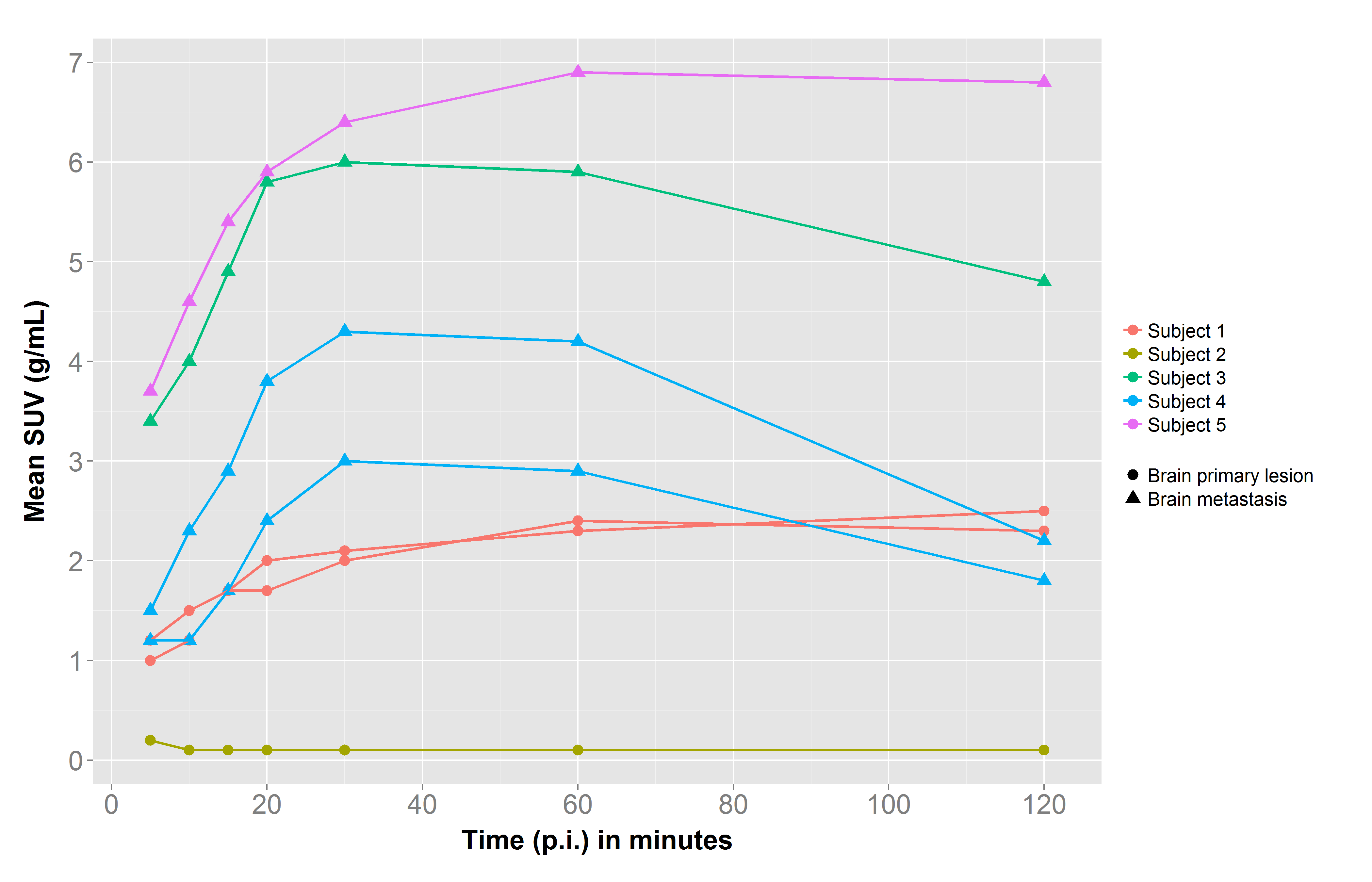

Supplement: S2 Fig — The brain lesions show a gradual increase in activity until 30 minutes then decreasing minimally. The only exception is Subject 2 with the low-grade oligodendroglioma who had no uptake. (TIFF) [file pone.0148628.s003.tiff]

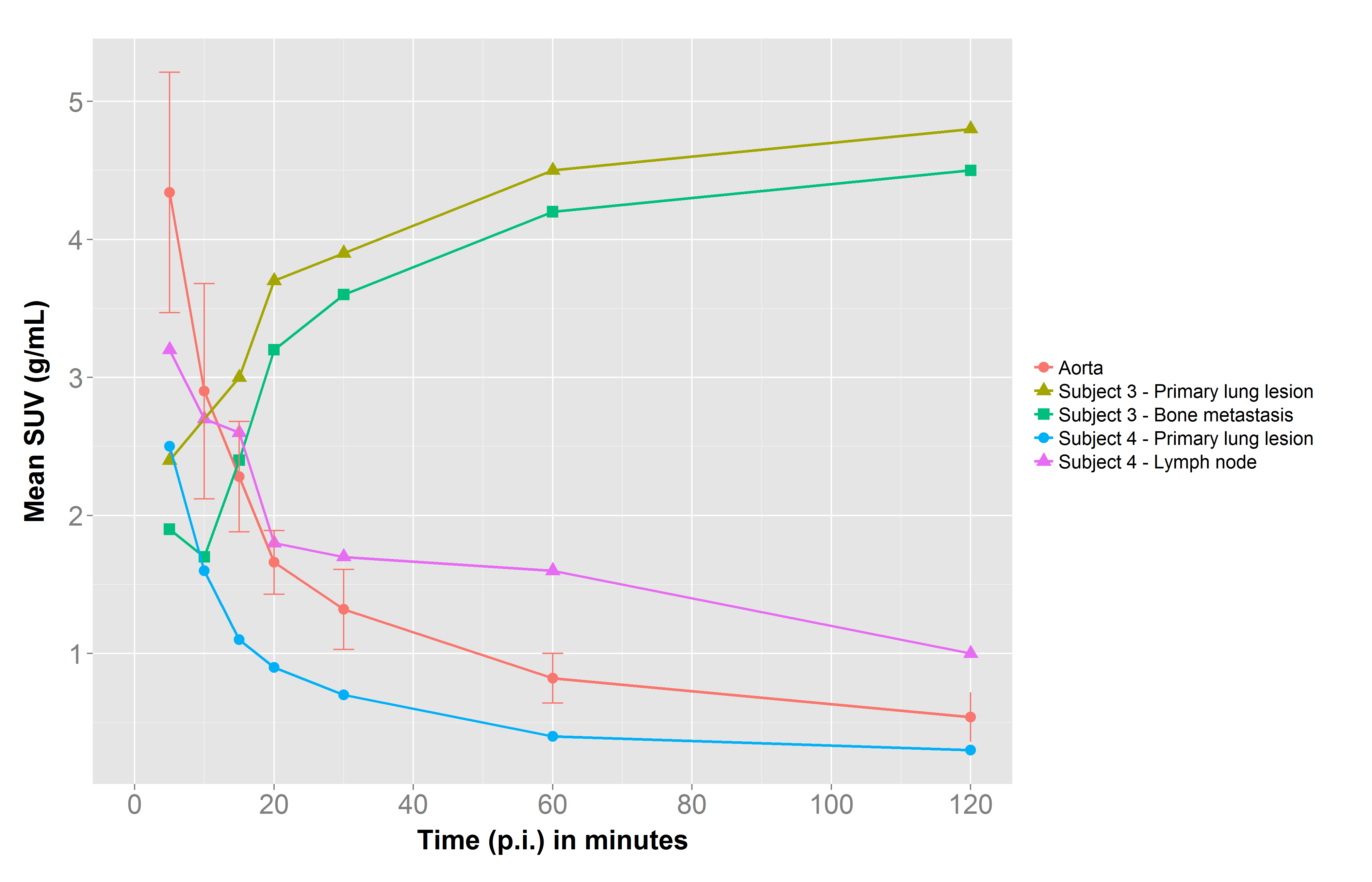

Supplement: S3 Fig — The active primary lung tumor and bone metastases for Subject 3 showed continually increasing activity over the time-course of imaging. The treated primary lung tumor and inflammatory lymph nodes for Subject 4 showed decreasing activity throughout the time-course of imaging. (TIFF) [file pone.0148628.s004.tiff]
