# Supplementary material for: Pilot Preclinical and Clinical Evaluation of (4S)-4-(3-[18F]Fluoropropyl)-L-Glutamate (18F-FSPG) for PET/CT Imaging of Intracranial Malignancies
Source: PLoS One. 2016 Feb 18;11(2):e0148628. doi: 10.1371/journal.pone.0148628 (PMC4758607; doi:10.1371/journal.pone.0148628)
Supplement: S1 Protocol — (PDF) [file pone.0148628.s005.pdf]

Synopsis

**Study title**	Open-label study for an exploration of tumor accumulation and safety and tolerability of the ^{18}F-labeled PET/CT (positron emission tomography / computed tomography) tracer BAY 94-9392 following a single intravenous administration of 300 MBq (corresponding to ≤ 100 µg total quantity) in patients with prostate cancer or other malignant tumors.
**Short title**	Tumor accumulation of BAY 94-9392 in patients with cancer.
**Clinical study phase**	1 (Proof of mechanism)
**Study objective(s)**	**All cancers**<br>1. Evaluation of the accumulation of BAY 94-9392 in primary cancer lesions confirmed by histology.<br>2. Evaluation of the accumulation of BAY 94-9392 in known or suspected local and/or systemic recurrent cancer lesions and/or lymph node metastasis or distant metastasis. If available: recurrent lesions or metastasis confirmed by histology/ biopsy.<br>3. Safety and tolerability of BAY 94-9392.<br>Note: Radiation dosimetry is studied in a different ongoing trial (BAY 94-9392/14207) in 5 healthy volunteers.<br>**Prostate cancer**<br>4. Assessment of the accumulation of BAY 94-9392 in histologically confirmed prostate tumor, benign prostate hyperplasia (BPH) or prostatitis following prostatectomy. If PET/CT or SPECT images from clinical routine are available: Comparison of these images with those obtained with BAY 94-9392.<br>**Non prostate cancer**<br>5. Comparison of BAY 94-9392 accumulation and [18F]-fluorodeoxyglucose (FDG) accumulation in histologically confirmed tumor lesions.
**Project code**	429100
**Test product(s)**	BAY 94-9392, ZK 6088248
**Name of active ingredient**	(S)- 4-(3-[^{18}F]Fluoropropyl)-L-glutamic acid
**Dose(s)**	Radioactive dose of 300 MBq (+/- 10%) of BAY 94-9392, corresponding to a total quantity of ≤ 100 µg
**Route of administration**	Intravenous bolus injection
**Duration of treatment**	One administration of a diagnostic agent
**Reference product(s)**	Not applicable
**Indication**	Diagnostic agent for initial staging, guidance for biopsy, detection of recurrence.

**Diagnosis and main criteria for inclusion**	Prostate cancer patients<br>1. Males ≥ 18 of age.<br>2. The primary cancer disease is histologically confirmed.<br>3. Patients with primary prostate cancer are scheduled for prostatectomy.<br>4. Patients with advanced primary or recurrent prostate cancer and a high likelihood to display lymph node metastasis are to be preferably included.<br><br>Other cancer patients<br>1. Males/females ≥ 18 years of age.<br>2. The primary cancer disease is histologically confirmed.<br>3. Cancer patient had an FDG PET/CT for detection, or staging, or restaging, or therapy response assessment that still showed tumor mass with high certainty, for melanoma, colorectal cancer or head and neck cancer. In case of malignant brain tumor or brain metastasis the FDG PET/CT from clinical routine is optional.
**Study design**	Open label, single-dose, explorative study in patients with histologically proven cancer and, preferably, tumor positive lesions in previously performed nuclear medicine imaging examinations.
**Methodology**	PET/CT imaging in patients: Visual and quantitative assessment of BAY 94-9392 PET/CT images and, for melanoma, colorectal cancer or head and neck cancer patients, intra-individual comparison with PET/CT images (since this an inclusion criterion) or for prostate cancer patients, malignant brain tumor patients or patients with brain metastasis, where available, intra-individual comparison with PET/CT images or SPECT images from clinical routine.
**Type of control**	Not applicable
**Planned study dates**	Start of study / recruitment: September 2010<br>End of recruitment: May 2011<br>End of study: June 2011
**Planned number of study centers / countries**	One center / United States
**Number of study subjects**	Total: Up to 30 patients. - Prostate cancer (total n=10, thereof 5 with primary prostate cancer, 5 with recurrent disease), melanoma (up to 5), colorectal cancer (up to 5), head and neck cancer (up to 5), malignant brain tumors or brain metastasis (up to 5)
	Minimum per center: N/A<br>Maximum per center: N/A
	Total number based on statistical rationale: N/A (explorative study)

**Primary variables**	PET/CT imaging in cancer patients: Visual assessment variables<br>• Overall image quality<br>• BAY 94-9392 overall lesions detection rate<br>• BAY 94-9392 lesion detection rate in comparison to histology, (applicable to patients with primary prostate cancer)<br>• BAY 94-9392 lesion detection rate in comparison to [^{18}F]-fluorodesoxyglucose (FDG) or, where available, SPECT, [^{11}C]- or [^{18}F]-choline, [^{11}C]-acetate<br>• BAY 94-9392 accumulation score in all lesions identified with this tracer.<br>• Comparative accumulation score BAY 94-9392 versus [^{18}F]-fluorodeoxyglucose (FDG) PET/CT or, where available, SPECT, [^{11}C]- or [^{18}F]-choline PET/CT, [^{11}C]-acetate PET/CT<br>• Impact of accumulation of BAY 94-9392 in other organs / body regions on visual assessment.
**Secondary variables**	Quantitative assessment<br>• SUV, SUVR<br>Safety variables<br>• Vital signs, laboratory findings, ECGs, AEs
**Plan for statistical analysis**	Visual and quantitative assessment of PET/CT images: Descriptive statistics, frequency tables<br>Safety: Descriptive statistics, frequency tables, intra-individual changes compared to baseline.<br>The study is explorative in nature.
