# Supplementary material for: Pilot Preclinical and Clinical Evaluation of (4S)-4-(3-[18F]Fluoropropyl)-L-Glutamate (18F-FSPG) for PET/CT Imaging of Intracranial Malignancies
Source: PLoS One. 2016 Feb 18;11(2):e0148628. doi: 10.1371/journal.pone.0148628 (PMC4758607; doi:10.1371/journal.pone.0148628)
Supplement: S1 Table — Significant differences are marked in bold. (DOCX) [file pone.0148628.s006.docx]

**S1 Table.** The comparison of the 18F-FSPG, 18F-FET and 18F-FDG uptake in blood, normal brain and brain tumor as well as analyses of tumor-to normal brain and tumor-to-blood ratios. Significant differences are marked in bold.

|  | **Kruskal-Wallis Test p-values** | **Pairwise multiple comparison** | | |
| --- | --- | --- | --- | --- |
|  |  | 18F-FET - 18F-FDG | 18F-FSPG – 18F-FDG | 18F-FSPG – 18F-FET |
| **Blood** | **0.007** | 0.412 | 0.282 | **0.005** |
| **Brain** | **0.008** | 0.412 | **0.007** | 0.150 |
| **Tumor** | **0.025** | 0.086 | 0.994 | **0.031** |
| **T/brain** | **0.012** | 0.674 | **0.015** | 0.097 |
| **T/blood** | **0.010** | 0.345 | 0.386 | **0.007** |
