# Supplementary material for: Pilot Preclinical and Clinical Evaluation of (4S)-4-(3-[18F]Fluoropropyl)-L-Glutamate (18F-FSPG) for PET/CT Imaging of Intracranial Malignancies
Source: PLoS One. 2016 Feb 18;11(2):e0148628. doi: 10.1371/journal.pone.0148628 (PMC4758607; doi:10.1371/journal.pone.0148628)
Supplement: S2 Table — (DOCX) [file pone.0148628.s007.docx]

**S2 Table.** Specific inclusion and exclusion criteria for enrollment in the human 18F-FSPG PET/CT imaging trial.

| **Inclusion Criteria** | **Exclusion Criteria** |
| --- | --- |
| - Males/females ≥ 18 years | - Concurrent severe and/or uncontrolled and/or unstable medical disease other than cancer or inflammation |
| - Females of no childbearing potential or females of childbearing potential but not pregnant or nursing. | - Known sensitivity to the study drug or components of the preparation |
| - Patient has a malignant brain tumor or brain metastasis. The primary tumor is histologically confirmed. | - Alcohol or drug dependence |
| - Adequate recovery (excluding alopecia) from previous surgery, radiation, and chemotherapy |  |
| - ECOG (Eastern Cooperative Oncology Group) performance status of 0-2, determined within one week prior to treatment with 18F-FSPG. |  |
| - Confirmation of adequate function of major organs and systems. |  |
| - No clinically relevant deviations in renal function as determined by Cockcroft and Gault method using serum creatinine at screening. |  |
| - No malfunction equivalent to CTC (Common toxicity criteria, CTCAE v3.0) toxicities grade > 2 of the liver (ALAT; bilirubin). |  |
| - Life expectancy of at least 3 months. |  |
