# Supplementary material for: Pilot Preclinical and Clinical Evaluation of (4S)-4-(3-[18F]Fluoropropyl)-L-Glutamate (18F-FSPG) for PET/CT Imaging of Intracranial Malignancies
Source: PLoS One. 2016 Feb 18;11(2):e0148628. doi: 10.1371/journal.pone.0148628 (PMC4758607; doi:10.1371/journal.pone.0148628)
Supplement: S3 Table — (DOCX) [file pone.0148628.s008.docx]

**S3 Table.** Average and standard deviation (S.D.) values for subjects’ vital signs, blood hematologies, and blood chemistries before injection of 18F-FSPG, after completion of imaging (approximately 3 hours post-injection), and the next day (approximately 24 hours post-injection).

|  | **Pre-injection** | **3 hrs post-injection** | **24 hrs post-injection** |  |
| --- | --- | --- | --- | --- |
|  | **mean (SD)** | **mean (SD)** | **mean (SD)** | **p-values** |
| **Vital Signs** |  |  |  |  |
| Systolic BP (mmHg) | 115.4 (5.7) | 136.2 (10.5) | 121.8 (12.9) | **0.03** |
| Diastolic BP (mmHg) | 75.2 (10.8) | 81.0 (10.1) | 76.6 (5.3) | 0.42 |
| Heart Rate (bpm) | 80.6 (17.1) | 74.4 (21.9) | 79.4 (11.2) | 0.09 |
| Temperature (^o^F) | 98.5 (0.4) | 98.0 (0.5) | 98.2 (0.4) | 0.19 |
| **Hematologies** |  |  |  |  |
| Hemoglobin (g/dL) | 13.7 (1.6) | 13.2 (1.5) | 13.8 (1.6) | 0.73 |
| Hematocrit (%) | 39.5 (4.6) | 38.3 (4.4) | 40.5 (4.8) | 0.62 |
| Platelets (K/µL) | 259.8 (74.1) | 235.6 (69.2) | 262.8 (77.1) | 0.83 |
| **Chemistries** |  |  |  |  |
| PT (sec) | 13.5 (1.0) | 13.6 (0.8) | 13.6 (1.2) | 0.89 |
| INR | 1.1 (0.1) | 1.1 (0.1) | 1.1 (0.1) | 0.60 |
| PTT (sec) | 29.4 (2.5) | 29.6 (2.0) | 29.7 (2.9) | 1.00 |
| Na (mmol/L) | 136.4 (2.3) | 137.6 (1.7) | 136.8 (3.5) | 0.74 |
| K (mmol/L) | 4.8 (0.8) | 4.0 (0.5) | 4.1 (0.9) | 0.34 |
| Cl- (mmol/L) | 101.6 (2.6) | 103.0 (2.3) | 102.8 (3.0) | 0.67 |
| CO2 (mmol/L) | 27.8 (3.0) | 27.6 (2.8) | 28.0 (1.9) | 0.97 |
| BUN (mg/dL) | 15.0 (5.0) | 14.6 (4.8) | 16.0 (5.5) | 0.91 |
| Creatinine (mg/dL) | 0.9 (0.3) | 0.9 (0.3) | 1.0 (0.3) | 0.92 |
| Anion Gap (mmol/L) | 7.0 (2.0) | 6.6 (1.8) | 6.0 (2.6) | 0.78 |
| Ca (mg/dL) | 9.3 (0.2) | 9.4 (0.2) | 9.3 (0.3) | 0.92 |
| Total Bili (mg/dL) | 0.5 (0) | 0.5 (0) | 0.5 (0) | 0.28 |
| AST (U/L) | 12.3 (4.0) | 18.2 (8.0) | 20.0 (9.3) | 0.49 |
| ALT (U/L) | 30.7 (5.7) | 37.2 (14.8) | 40.2 (15.5) | 0.70 |
| ALK (U/L) | 121.8 (35.3) | 118.4 (29.1) | 120.2 (23.9) | 0.98 |
| Albumin (g/dL) | 3.6 (0.1) | 3.5 (0.3) | 3.7 (0.2) | 0.61 |
| Protein Total (g/dL) | 7.5 (1.0) | 7.1 (0.7) | 7.4 (0.8) | 0.87 |
| Globulin (g/dL) | 3.9 (1.0) | 3.6 (0.5) | 3.7 (0.6) | 0.89 |
